# Surgical Membranes as Directional Delivery Devices to Generate Tissue: Testing in an Ovine Critical Sized Defect Model

**DOI:** 10.1371/journal.pone.0028702

**Published:** 2011-12-12

**Authors:** Melissa L. Knothe Tate, Hana Chang, Shannon R. Moore, Ulf R. Knothe

**Affiliations:** 1 Department of Biomedical Engineering, Case Western Reserve University, Cleveland, Ohio, United States of America; 2 Department of Mechanical & Aerospace Engineering, Case Western Reserve University, Cleveland, Ohio, United States of America; 3 Department of Orthopaedic Surgery, Cleveland Clinic, Cleveland, Ohio, United States of America; Instituto de Engenharia Biomédica, University of Porto, Portugal

## Abstract

**Purpose:**

Pluripotent cells residing in the periosteum, a bi-layered membrane enveloping all bones, exhibit a remarkable regenerative capacity to fill in critical sized defects of the ovine femur within two weeks of treatment. Harnessing the regenerative power of the periosteum appears to be limited only by the amount of healthy periosteum available. Here we use a substitute periosteum, a delivery device *cum* implant, to test the hypothesis that directional delivery of endogenous periosteal factors enhances bone defect healing.

**Methods:**

Newly adapted surgical protocols were used to create critical sized, middiaphyseal femur defects in four groups of five skeletally mature Swiss alpine sheep. Each group was treated using a periosteum substitute for the controlled addition of periosteal factors including the presence of collagen in the periosteum (Group 1), periosteum derived cells (Group 2), and autogenic periosteal strips (Group 3). Control group animals were treated with an isotropic elastomer membrane alone. We hypothesized that periosteal substitute membranes incorporating the most periosteal factors would show superior defect infilling compared to substitute membranes integrating fewer factors (i.e. Group 3>Group 2>Group 1>Control).

**Results:**

Based on micro-computed tomography data, bone defects enveloped by substitute periosteum enabling directional delivery of periosteal factors exhibit superior bony bridging compared to those sheathed with isotropic membrane controls (Group 3>Group 2>Group 1, Control). Quantitative histological analysis shows significantly increased *de novo* tissue generation with delivery of periosteal factors, compared to the substitute periosteum containing a collagen membrane alone (Group 1) as well as compared to the isotropic control membrane. Greatest tissue generation and maximal defect bridging was observed when autologous periosteal transplant strips were included in the periosteum substitute.

**Conclusion:**

Periosteum-derived cells as well as other factors intrinsic to periosteum play a key role for infilling of critical sized defects.

## Introduction

Critical sized defects do not heal spontaneously without surgical intervention. Numerous surgical techniques have been employed to treat these defects with limited success and a large number of complications [1]. Distraction osteogenesis has become a standard of care for the treatment of large diaphyseal bone defects due to superior union rates achieved with it in comparison with other surgical techniques [1]–[19]. Nonetheless, distraction osteogenesis has several disadvantages including long and labor-intensive treatment times, significant demands on patient compliance, discomfort, and high rates of complications with associated requirements for multiple surgical procedures following the index procedure. In addition, the technique requires significant technical expertise, which limits the number of orthopaedic surgeons with the training and experience necessary to perform the procedure. Even when implemented by surgeons with significant expertise, the relatively high rate of complications and subsequent need for reoperations associated with the technique persists [11], [13], [17].

**Figure 1:**
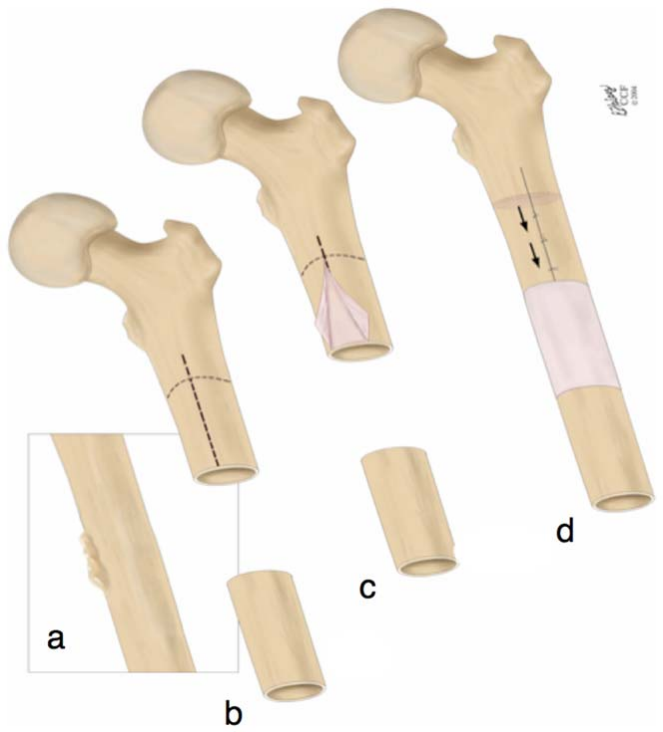
The one-stage bone-transport procedure [20]–[22] harnesses the regenerative potential of the periosteum and cells therein, *e.g.* for treatment of a critical sized long bone defect after tumor resection. (**a**) The tumor is first identified and (**b**) then resected *in toto*, leaving a critical sized defect. The periosteum is scored (dashed line, **b**) and (**c**) gently peeled back off the proximal bone, leaving denuded bone below. (**d**) The denuded bone is osteotomized and transported distally to fill the defect zone. It is docked to the distal, healthy femur using ligament sutures. A limiting factor in implementation of this technique is the availability of periosteum in areas easily accessible to the surgical site. This is particularly problematic in high impact trauma injuries such as blast wounds or high speed sports or traffic injuries. Images used with permission [21].

These and other factors provided the impetus for the development of a one stage bone transport procedure that harnesses the regenerative power of the periosteum to fill in critical sized defects without the need for adjuvant bone graft (**Fig. 1**) [20]–[22]. Histology and quantitative micro-computed tomography (μ-CT) studies indicate that the cells and blood supply within the periosteum are key to success of the one stage procedure [21], [22]. Interestingly, filling of the periosteum enveloped defect with autologous bone graft from the iliac crest retards the infilling of the defect due to the need for prior osteoclastic resorption [22]. Based on experimental and several clinical cases, implementation of the one stage bone transport procedure appears to be limited only by the amount of healthy periosteum available [20]–[22]. The current study addresses that limitation.

In the current study we implement a newly developed surgical reconstruction membrane as a substitute for the periosteum to treat a critical sized (2.54 cm) defect in a previously developed adult ovine femur model with intramedullary nailing for mechanical stabilization [21]. Our goal was to design a periosteum substitute that mimics the structure-function properties inherent to native periosteum. As a bi-layered membrane, native periosteum exhibits anisotropic, composite structure conferring unique functional properties [23]. The periosteum's outer layer comprises mostly collagens, aligned with the longitudinal axis, and elastin; this outer layer is hypothesized to control bone shape/length during growth [24], to contribute to bone toughness, and to limit displacement of fracture fragments to stabilize bones after bone failure [25]. The cambium, or periosteum's innermost layer, comprises mostly cells that appose the underlying bone [26]. The progenitor cells within the inner cambial layer are responsible for continual periosteal bone apposition during life and confer regenerative properties to the periosteum [21], [22], [27]–[30].

**Figure 2:**
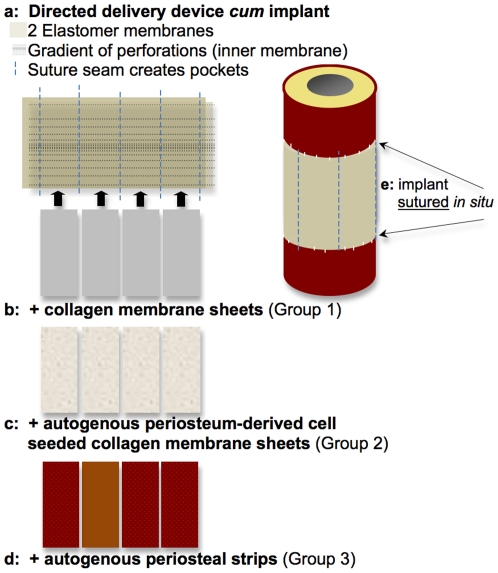
A periosteal substitute, novel directed delivery device *cum* implant was developed and implemented to mimic structure-function relationships intrinisic to the periosteum and to enable vectorial delivery, *i.e.* control of delivery direction and magnitude or concentration, of periosteal factors. In the current study we tested the efficacy of directional delivery of periosteal factors to enhance defect healing. (**A**) The implant comprises an outer elastomeric membrane (FDA approved material), an inner elastomeric membrane with a gradient of perforations of highest concentration furthest from the proximal and distal edges of the defect zone. The layers are sewn together with suture material, creating four pockets into which periosteal factors can be tucked. (**B**) Collagen membranes (FDA approved) were cut and tucked into the pockets of Group 1 implants. (**c**) Cells were isolated from periosteum of the bone removed to create the defect, seeded on the collagen membranes (FDA approved), and tucked into the pockets of Group 2 implants. (**D**) Strips of periosteum were resected from bone removed to create the defect and tucked into the pockets of Group 3 implants. Bony bridging of defects sheathed by each respective periosteum substitute was compared between groups and with a Control group implementing an isotropic, simple elastomeric membrane (FDA approved). (**E**) The periosteum substitute implant was then sutured to the proximal and distal edges of periosteum lifted along the edges of the defect and sutured close along the longitudinal axis of the lateral aspect. Refer to **Fig. 3** for intraoperative photo.

Hence, we designed our periosteum substitute to serve as a delivery device *cum* implant exhibiting a modular design with pockets to allow for directional (outside→in), spatial (via anterior, posterior, medial and lateral pockets), and temporal control of factor delivery (**Fig. 2A**). The periosteum substitute design *per se* mimics the structure of native periosteum, the outer layer of which is made up of the structural proteins elastin and collagen. In this study, we used the periosteum substitute for the controlled addition of periosteal factors including the presence of collagen, the predominant structural protein of the extracellular matrix that is present in the outer sheath of the periosteum [26] (Group 1, **Fig. 2B**), cells residing within the periosteum (Group 2, **Fig. 2C**), and autologous periosteal strips (Group 3, **Fig. 2D**). Animals of the Control group were treated with an isotropic membrane alone. We hypothesized that periosteal substitute membranes incorporating the most periosteal factors would show superior defect infilling compared to substitute membranes integrating fewer factors (*i.e.* Group 3>Group 2>Group 1>Control).

## Methods

### Ethics Statement

All work was conducted according to relevant national and international guidelines; full details of the study were approved by the Amt für Lebensmittelsicherheit und Tiergesundheit Graubünden (Institutional Review Board of the Canton of Grisons, Switzerland), Tierversuchsbewilligung (Animal Experiment Permission) No. 15/2008.

### Overview

Previously described surgical protocols were used to create critical sized, middiaphyseal femur defects [21], first in one short term (3 week) pilot group treated with periosteum substitute implants incorporating collagen membranes (Group 1). Thereafter, we treated four groups of five skeletally mature Swiss alpine sheep for sixteen weeks, a period over which defects surrounded by periosteum *in situ* heal completely and untreated defects do not heal (providing proof of critical size) [21], [22].

Each group was treated using a substitute periosteal membrane designed and manufactured according to our protocols [31], [32] to deliver specific factors to the defect zone (**Fig. 2**, Table 1). The substitute periosteum implants comprised combinations of FDA-approved materials and/or autologous materials including periosteum derived cells and periosteal strips from the bone removed to create the defect. The FDA approved materials used to make the implants included medical grade silicone elastomer sheeting, absorbable collagen membrane derived from bovine achilles tendon, and nonresorbable sutures (**Fig. 2**). The periosteum substitutes were designed to be easily manufactured, modular (the pocket design allows for substitution as well as precise localization and timing for release of specific factors or combinations of factors), fully sterilizable with other surgical armamentaria, and easy to use by surgeons with varying degrees of expertise.

**Table 1:** Defect bridging was scaled to assess objectively defect bridging without the ability to measure volume of bone generated quantitatively due to the presence of the intramedullary nail.

GROUPS	0–25%	25–50%	50–75%	75–100%
EMPTY	7/7			
ControlMembrane	4/5	1/5		
1: Membrane + Collagen	5/5			
2: Membrane + Collagen + Cells	1/5	2/5	1/5	1/5
3: Membrane + PeriostealStrips		2/5	1/5	2/5
INTACT PERIOSTEUM				7/7

For comparative purposes, images from a previous study using the same animal model were assessed using the same scaling method, comparing to the empty defect zone (see **Fig. 7A**), which was left without treatment and which never healed, indicative of a true critical sized defect [21]. Also, the use of intact periosteum with adherent cortical bone chips on the inner surface, from the previous study, is compared as the “best observed outcome” which provides target specifications for the periosteum substitute implant [21].

To create the modular design, an inner membrane was first perforated with a gradient of holes along its entire length, with the highest concentration of holes near the center of the defect region, decreasing toward the edges that are sutured to the periosteum of healthy bone proximal and distal to the defect zone. The pattern of holes was achieved using a double (parallel) sewing machine needle (without thread) and setting the sewing machine stitch length to achieve equidistance at the center of the defect between rows of double holes. The inner membrane was then sewn, using the suture material, to the outer membrane, which was devoid of perforations, creating a long sleeve (3.5×10 cm) with four, 2 cm wide pockets (**Fig. 2A**). The whole construct was then placed on surgical dressing gauze and encapsulated within a sterilization sleeve for steam sterilization in an autoclave with other surgical instruments.

**Figure 3:**
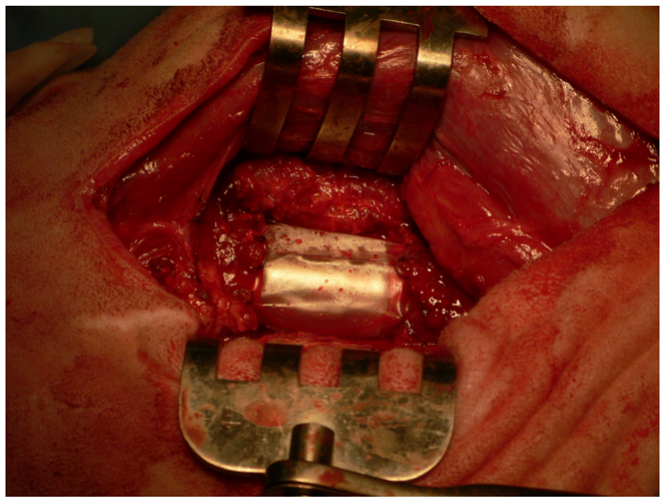
Intraoperative photo showing a periosteal substitute *in situ*, enveloping a critical sized defect in the middiaphysis of the ovine femur.

Shortly before surgical implantation (in the sterile surgical operating theatre), the substitute periosteum implant was removed from the sterile packaging and experimental (periosteal) factors were placed in the implant pockets (collagen membrane - Group 1, collagen membrane seeded with autogenous periosteum derived cells - Group 2, or strips of autogenous periosteum transplants from bone removed to create defect - Group 3, Table 1). The implant was then sutured to periosteum lifted along the edge of the remaining bone proximal and distal to the defect (**Fig. 2D** **,** **Fig. 3**). The control group was treated with a simple, isotropic silicone elastomer membrane (without flow directing architecture) around the critical sized defect. Group 1 was treated with a membrane and collagen sheets, incorporating flow directing architecture, which allows for directional transport, *i.e.* from the proximal and distal edges axially toward the center, and from the membrane radially toward the intramedullary nail. Group 2 received the membrane as in Group 1, but the collagen sheet within the membrane was pre-seeded with cells isolated from periosteum of the autologous defect bone and incubated at 37°C overnight. After careful aspiration and washing, the cell-seeded membrane was then placed into the four pockets of the substitute periosteum. Finally, Group 3 received the membrane in combination with autologous periosteal transplant strips (isolated from the bone removed to create the defect zone) in place of the collagen sheets.

Animals were euthanized at 3 (pilot group) and 16 weeks after surgery. Femoral tissue blocks including surrounding musculature were scanned with the intramedullary (IM) nail *in situ* using high resolution μ-CT to determine efficacy of the periosteal substitutes for defect bridging. Due to the retention of the steel IM nail (which can cause imaging artifacts due to beam hardening), it was not possible to obtain volumetric data from the μ-CT images. For this reason, bridging of the defect was ranked, by a blinded observer, on a quartile scale (**Table 1**, 0–25%, 25–50%, 50–75%, 75–100%), indicating degree of defect coverage for each treated sample in each experimental group.

Finally, quantitative histomorphometry was carried out to measure bone generation in the defect zone and to compare the mean bone regenerated between groups.

### Detailed Methods

#### Sheep and surgical procedures

The experimental protocol was carried out, using skeletally mature Swiss alpine sheep (*n* = 5 per group, 3 experimental and one control group), at the AO Research Institute in Davos, Switzerland, with the approval of the animal care and use commission of the Canton of Grisons.

All surgeries were performed by URK. Surgery was performed with sheep in the right lateral position. After general anaesthesia with intubation and spinal anaesthesia, the left hindlimb was shaved and prepped with Hibiscrub solution and draped. The right hind limb was strapped with a belt extending around the operating table and the abdomen. The sheep was then transported into the operating room and the left hind limb was prepped and draped in the usual sterile fashion. A lateral parapatellar approach was performed to enter the knee joint to prepare for retrograde IM nailing. The patella was dislocated medially. A 4 mm entry hole was then placed in the midline of the trochlea, approximately 8 mm anterior to the intracondylar notch. Sequential reaming of the intramedullary canal was performed with increasing sized cutting reamers, starting at 7 mm, going up to 14 mm. The flexible Synthes reamer system was then used to ream 15 mm, up to 16 mm, in 0.5 mm increments.

Attention was then turned lateral to the femur and a second, approximately 12.5 cm incision was made lateral over the femur. The *vastus lateralis fasciae* and the intramuscular plane were developed to expose the lateral aspect of the femur. Meticulous hemostasis and ligation of larger vessels was performed, distal third of the femur. The *vastus lateralis* was then detached from the bone with a scalpel to expose the distal femur with a 1–2 mm layer of muscle attached. A 2.54 cm critical sized defect was then created 8 cm proximal to the knee joint line with an oscillating saw using small amplitude and preserving the soft tissue with rounded custom retractors.

The periosteum was then lifted circumferentially, approximately 5 mm from the proximal and distal defect/osteotomy edges, with a periosteal elevator. Thereafter, the intramedullary nail was inserted retrograde and care was taken to maintain the 2.5 cm critical defect size. Two proximal and two distal 4.9 mm interlocking bolts were then placed.

The 3.5 cm periosteum substitute membrane was then introduced around the defect using a custom S-shaped retractor to maintain space medial to the femur-nail. The membrane was placed from anterolateral side and brought posterior around the defect. The membrane was designed to overlap 0.5 cm distal and proximal to the defect. In the case of the experimental membrane, the outer layer overlapped the elevated periosteum by 5 mm and the inner layer was placed so that the periosteum was sandwiched between and sutured carefully in place with Dermalon 4.0 sutures, while applying inner and outer rotation of the hind limb as needed to reach regions behind the IM nail. In the case of the simple control membrane, the membrane was placed overlapping the elevated periosteum by 5 mm and sutured in place. The edges of the membrane were connected laterally using Dermalon 4.0 sutures and trimmed to fit the circumference of the defect.

The knee and lateral femoral incisions were then closed in layers with no suction tube trains, using vicryl #1 for fascia, 2.0 for subcutaneous, and Moncryl 3.0 for subcuticular running sutures.

#### Periosteum substitute implant manufacture

Substitute periosteal membrane were designed and manufactured by MKT as sterilizable, modular units to deliver specific factors to the defect zone. Delivery devices (modular implant with pockets) were manufactured from FDA approved materials, including medical grade silicone sheeting (Bioplexus, 0.005″ thick medium durometer silicone elastomer, Ventura, CA), and nonresorbable sutures (Dermalon 4-0, monofilament nylon, Syneture, Covidien Surgical, Dublin, Ireland). Absorbable collagen membranes (Biomend®, derived from bovine achilles tendon, Zimmer Dental, Carlsbad, CA), designed for periodontal use, were cut to size and placed in implant pockets in Groups 1 (collagen membrane alone) and 2 (collagen membrane seeded with cells isolated from autologous periosteum). Autologous periosteal strips were placed in the pockets of implants from Group 3, retaining the anatomic orientation of the inner and outer surfaces. Cut to size but otherwise unaltered, isotropic medical grade silicone sheets were used in the control group. Bone growth within implants incorporating increasing numbers of periosteal factors were compared with control implants as well as with the baseline critical sized defect control (untreated) and “gold standard” *in situ* periosteum treated defects studied previously [21].

#### Cell culture methods - isolation and proliferation of periosteum derived cells

Cell culture was carried out by HC. Ovine periosteum explants resected from the bone removed to create the defect were immediately placed in TBSS with 1% Penicillin/Streptomycin (P/S). The explants were then minced and placed in spinner flasks with high glucose DMEM (GIBCO, Grand Island, NY) with 1% P/S and 0.3% Collagenase II (Worthington Biochemical Corporation, Lakewood, NJ). This was incubated for 3 hours in a 37°C incubator in 95% humidified air and 5% CO_2_. The cells were filtered through a 40 mm vacuum filter to remove fibrous tissue, centrifuged to remove the collagenase solution, and resuspended in high glucose DMEM with 10% FBS and 1% P/S. The cells were then seeded on precut collagen membranes, which were sized to fit into the periosteal implant pockets. The seeded membranes were then incubated overnight at 37°C, in 95% humidified air with 5% CO_2_. The next morning, after careful aspiration and rinsing in TBSS to remove any cell culture chemicals, the seeded membranes were then placed into the four pockets of the substitute periosteum implant, under sterile conditions, in the surgical theater.

Prior to the surgical study we characterized growth rates of the ovine periosteum derived cells. Adherent periosteum derived cells were plated at 34,000 cells/cm^2^ high sets of 8 in 96-well plates in high glucose DMEM with 10% FBS and 1% P/S. Cell culture media was changed every 2–3 days. Cell numbers were analyzed for 7 days using WST-1 reagent (Roche Applied Science, Indianapolis, IN) and incubated for 4 hours before measuring absorbance.

#### Undecalcified histological preparation and measurements of tissue generation

Histological sectioning and analysis was carried out by SM and MKT. Following resection and μ-CT imaging, the femur and surrounding tissue were fixed and embedded in poly(methyl methacrylate) (PMMA) for undecalcified histology processing. A diamond wire saw was used to produce three transverse sections through the tissue block with intramedullary nail *in situ;* sections were taken at intervals of approximately 6.5 mm to span the entire defect (Well Precision Diamond Wire Saw 6234, Norcross, GA), and polished with a variable speed Grinder-Polisher (Buehler EcoMet 4000, Lake Bluff, IL). Both sides of each section were imaged, and full collages of the cross-sections were created first, at 1.6× magnification to establish a qualitative perspective on *de novo* bone generation and healing trends between and across groups. Thereafter, cut surfaces were stained with Giemsa and Eosin to quantify new tissue area and distribution. The stain colors cell nuclei and connective tissue dark blue, and mineralized tissue (bone) pink. Collages were made of the proximal and distal side of all slices at 5× magnification using an inverted epifluorescent microscope with automated, computerized stage, (Leica DMIRE2, Wetzlar, Germany) and using broad spectrum UV excitation to quantify *de novo* bone generation and compare areas of new bone generated between groups. Whole collages were processed using a custom designed algorithm to identify and segment out mineralized (pink) and cartilage (blue) tissue (Adobe Photoshop CS5, Adobe Systems Incorporated, San Jose, CA). Thereafter, total areas of regenerates for mineralized and cartilage tissue were calculated using a pixel-to-area scaling factor (Image J version 10.2, NIH, Bethesda, MD).

To summarize the sample size for histological measures, three sections were made through the mid-diaphyseal region of each defect site, resulting in a total of six surfaces (both proximal and distal) for each animal. With five animals in each group, this resulted in a total of 30 high-resolution collages for each group (Control, Group 1, Group 2 and Group 3), *i.e.* 120 collages in total. Three collages were excluded because due to their near proximity to the proximal or distal bone outside of the defect zone. This resulted in an *n≥*27 collages for all groups.

## Results

**Figure 4:**
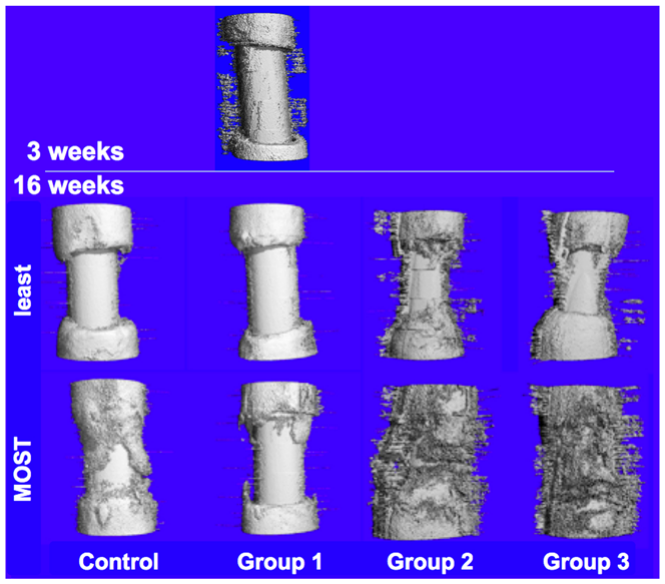
High resolution micro-computed tomography (μ-CT) of bone regenerate in Group 1 at 3 weeks and in all groups at 16 weeks after surgery. The intramedullary nail is present along the longitudinal axis of all specimens. Each group comprised five sheep and the sample images showing the least and most amount of new bone are depicted for each group.

**Figure 5:**
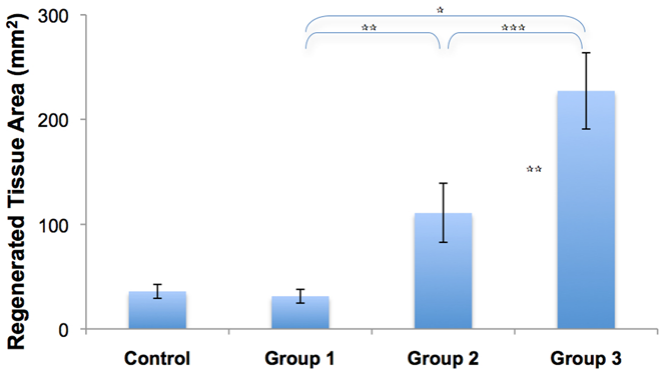
Mean area of *de novo* bone regenerate in defect zone, measured in histological sections through the defect zone. Error bars represent standard error of the mean. Statistical significance of differences between groups was tested using the Mann-Whitney (Wilcoxon) test, with significance defined as p<0.05. ^
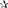
^p = 0.0003, ^
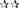
^p = 0.0459, ^
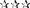
^p = 0.037.

Histomorphometric and μ-CT data showed that membranes incorporating periosteal factors significantly improved bone generation in the critical sized defect compared to isotropic, unstructured control membranes made of the same material (Group 3>Group 2>Control, Table 1, **Fig. 4** **,** **Fig. 5** **, Fig. S1, S2, S3, S4**). Bone generation in defects treated with isotropic control membranes appeared to emanate from the proximal and distal periosteal edges of the defect zone, tapering along the surface of the intramedullary nail, from the intact bone toward the center of the defect (**Fig. S1**); more new bone was observed in the proximal half of the defect than the distal half and limited contact was observed between the two areas of bone ingression. In addition, limited proliferative woven bone was observed to radiate inward from the isotropic membrane in only one specimen treated with the isotropic control membrane (9047, **Fig. S1**).

At three weeks after treatment with periosteum substitute implants incorporating collagen sheets (Group 1), intramembranous, woven bone was observed emanating from the implant toward the intramedullary nail in four of five defects studied (**Fig. 4** **, Fig. S2A**). At three weeks after surgery, almost no ingression of bone was observed from the intact periosteum of the proximal and distal edges. The intramembranous bone observed at three weeks was no longer evident in μ-CT images at sixteen weeks (**Fig. S2B**). In fact, at 16 weeks, Group 1 did not show evidence of improved defect filling compared to the isotropic control membrane, and the best case of the Control membrane showed more infilling than the best case of the implants incorporating collagen sheets alone. Furthermore, in contrast to the control group, less ingression of bone was observed from the proximal and distal edges of the defect. Bone that did ingress proximally and distally appeared to exhibit less tapering toward the intramedullary nail than in the Control group.

**Figure 6:**
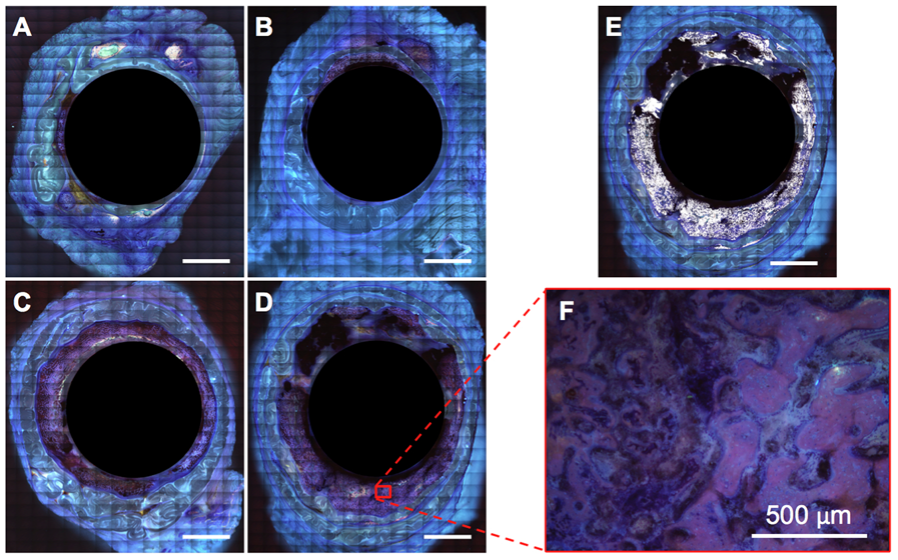
Histological cross-sections for Giemsa and eosin-stained specimens showing representative areas of tissue regeneration for each control and experimental group. (**A**) Stained and unprocessed specimen for the Control Group. (**B**) Stained and unprocessed specimen for Group 1 incorporating collagen sheets. (**C**) Stained and unprocessed specimen for Group 2 incorporating periosteum derived cells seeded on collagen sheets. (**D**) Stained and unprocessed specimen for Group 3 incorporating periosteal strips. (**E**) Segmented image from Group 3 (**D**) highlighting mineralized tissue. (**F**) Demonstration of collage resolution, as shown in a single field-of-view (acquired from **D**). Scale bar for (**A**–**E**) is 5 mm.

Insertion of collagen sheets seeded with periosteal derived cells (Group 2) within the periosteum substitute implant resulted in significantly increased bone generation (**Fig. 5** **,** **Fig. 6**) and defect bridging (**Fig. 4** **, Fig. S3**) compared to the isotropic control membrane (Control) as well as compared to the periosteum substitute with collagen sheets alone (Group 1). In contrast to both the Control and Group 1 at 16 weeks, woven intramembranous bone was evident in μ-CT images from Group 2 at 16 weeks. In addition, bone infilling appeared to have ingressed from the proximal and distal edges of the defect, in nearest proximity to healthy bone (with a tendency toward more bone in the proximal than the distal half of the defect). In one specimen exhibiting the most robust infilling/healing response of all in Group 2, the inwardly radiating intramembranous bone also coalesced most with proximally and distally ingressing bone, compared to other specimens within the group (9046, **Fig. S3**).

Maximal bone infilling of the defect was observed in Group 3, where autologous strips of periosteum were tucked into the pockets of the modular periosteum substitute (**Fig. 4**, **Fig. 5** **,** **Fig. 6** **, Fig. S4**). Two specimens exhibited coalescence of intramembranous bone emanating from the membrane and bone ingressing from the proximal and distal edges of the defect (9049, 9050, **Fig. S4**). Two specimens exhibited a weaker intramembranous proliferative bone response and ingression from the proximal, respectively distal, edges (9048, 9051, **Fig. S4**) and one exhibited an intermediate response (9052, **Fig. S4**).

**Figure 7:**
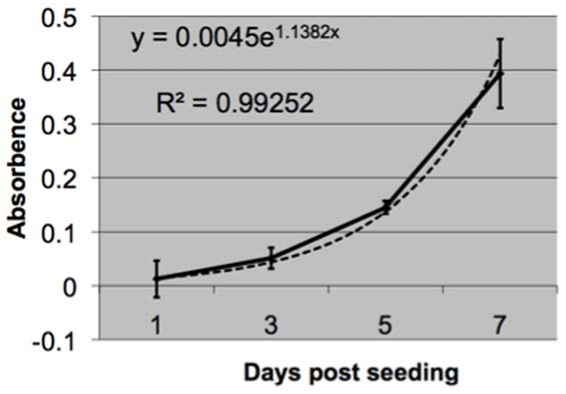
Proliferation of seeded cells derived from ovine periosteum. Error bars depict standard deviations.

Periosteum-derived cells proliferate exponentially during the first seven days of seeding; linear regression gives the following equation to predict cell number (y) as a function of days (x):
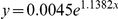
Although the cell proliferation studies could not be conducted on the collagen membrane due to the colorimetric measurements needed for the proliferation assay, proliferation data shows that the isolated cells are viable and proliferate steadily over the course of 7 days (**Fig. 7**).

## Discussion

The current set of experiments indicate a key role for directional delivery of periosteum-derived cells as well as other factors intrinsic to periosteal transplants for infilling of critical sized defects. Based on quantitive histomorphometry measurements as well as micro-computed tomography data, bone defects enveloped by substitute periosteum enabling directional delivery of periosteal factors show significantly more bone generation as well as superior bony bridging compared to those sheathed with isotropic elastomer membrane controls. Maximal bridging was observed when autologous periosteal transplant strips were included in the periosteum substitute, followed by the group in which the periosteum substitute delivered cells (derived from the periosteum), radially, to the defect. Whereas the periosteum substitute incorporating collagen membranes showed evidence for woven bone generation, radially inward from the inner surface of the implant, at three weeks after surgery, the radial woven bone generation was not evident at sixteen weeks after surgery. Similar to the isotropic control membrane, bone generation in the defect zone at sixteen weeks after surgery occurred mainly via bone ingression from proximal and distal edges of intact bone and periosteum.

**Figure 8:**
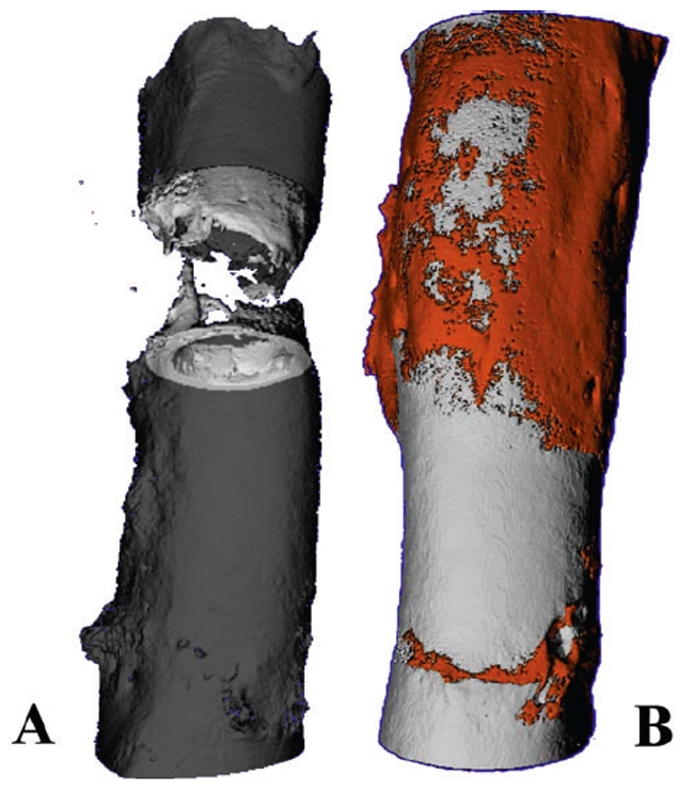
High resolution micro-computed tomograph (μ-CT) images of healing 16 weeks after critical sized defect from the previous study [**21**] (used with permission). (A) Baseline, untreated control defect, confirming critical size of defects (does not heal without treatment). Defect is completely healed in experimental groups, e.g. (B) including group treated with periosteum, left *in situ* with small cortical bone chips adherent to inner surface, around defect.

To place the results of the current study in context with our previous work on treatment of criticial sized bone defects in the same ovine model, we compared our current results with the those of the baseline, untreated control as well as those of the “best outcomes group” where the periosteum (with adherent cortical bone on its inner surface) was left *in situ* around the defect (**Fig. 7**) [21]. Due to the necessity to retain the intramedullary nail in the femoral blocks of the current study, μ-CT observations were compared using a defect coverage scale. Thereafter we used histomorphometric measures to compare quantitatively bone generated *de novo* in the defect zone. In the previous study, the baseline control specimens were left empty; the lack of infilling and bridging of the defect in all five specimens of this group provided evidence for the defect's critical size (**Fig. 8A**). Both the Control group and Group 1 of the current study showed similar results, with no bridging of the defect at 16 weeks after surgery. Thus, neither implementation of the isotropic control membrane nor use of the directional delivery membrane incorporating a collagen sheet showed improvement in healing compared to the untreated defect of the previous study. In contrast, Groups 2 and 3 of the current study, which implemented the directional delivery membrane in conjunction with periosteum-derived cells seeded on the collagen sheet and strips of endogenous periosteum, respectively, showed superior defect bridging and bone generation in the defect zone compared to the untreated control of the previous study. Accounting for all five specimens of each respective group, healing was not as robust as that observed in the best outcomes group of the previous study, where five of five defects completely bridged (**Fig. 8B**) [21].

Given our explicit goal to mimic structure-function relationships in the design of our periosteum substitute membrane, we were surprised that inclusion of collagen sheets within the elastomeric modular pockets showed no significant improvement in bone generated within or bridging of the defect zone, compared to isotropic membrane controls. At 16 weeks after surgery, previous studies showed all critical sized defects to have healed completely when surrounded by periosteum *in situ* [21]. Hence, based on quantitative histomorphometric and qualitative μ-CT data of the current study, it appears the delivery of cells (seeded on collagen sheets), or in combination with periosteum intrinsic factors, provides a much more potent stimulus for tissue building than delivery of collagen alone. Using this previously tested ovine defect model with mechanical stabilization via a solid intramedullary nail, the medullary niche and its resident, multipotent marrow stromal cells are removed at the time of surgery. In native periosteum, collagen and elastin form the outer, fibrous sheath, encompassing the cambium (cellular) layer. When left *in situ* around the defect, periosteum derived cells egress from the cambium layer, inwards, to fill the defect with intramembranous bone [21], [22]. Of particular note, proliferation of periosteum derived cells cultured on tissue culture styrene does not appear to be contact inhibited, in contrast to other pluripotent cell lines [33]. Surprisingly little reference data exists with regard to characterization of periosteum derived multipotent cells in sheep, likely due to the paucity of ovine surface markers (for FACS) or rtPCR primers. In future studies it will be interesting to better mimic molecular and cellular interactions to further functionalize the periosteum substitute membrane.

From a clinical perspective, it would be expected that, in surgical cases where insufficient periosteum is available to suture *in situ* around tissue defects, use of the directional delivery implant in conjunction with periosteum strips harvested from other areas would provide a superior alternative to treatment with a simple isotropic membrane sleeve. However, harvesting of periosteum strips from elsewhere (*e.g.* proximal or distal to the area of tumor resection or trauma) was not tested directly in the current study and potentially could be associated with other complications when implemented in patients. In the current study, periosteal strips were resected from the bone to create the defect. Inclusion of autologous periosteal strips would be expected to yield superior results to inclusion of autologous periosteum-derived cells for similar reasons, given the necessity of harvesting tissue and subjecting the patient to a two stage procedure in order to harvest and proliferate autologous cells on collagen membranes for implantation in the directional delivery device. However, another potential scenario would be to pre-seed collagen membranes with pluripotent cells from a stem cell bank (*e.g.* [34], [35]) prior to surgery and then to incorporate the pre-seeded collagen sheets into the directional delivery membrane at the time of surgery. This clinical scenario would not only avoid the necessity of a two stage procedure, but it would also not involve additional harvesting of tissue (and potential additional complications associated with more extensive surgical approaches) from the patient.

The current study implicates different spatiotemporal patterns of bone formation via bone induction and bone conduction that are modulated differently, depending on the surgical membrane used. Whereas use of the directional delivery membrane appeared to enable (rapid) early bone induction, radially inward from the implant to the surface of the intramedullary nail, both the isotropic and directional delivery membranes appeared to serve as an osteoconductive sleeve as well, allowing for ingression of bone from the proximal and distal edges of the defect, axially into the defect zone. Albrektsson and Johansson differentiate osteo*induction*, “the recruitment of immature cells and stimulation of these cells to develop into preosteoblasts”, from osteo*conduction* or surface mediated bone growth [36]. Albrektsson and Johansson's definition somewhat contrasts with earlier descriptions of osteoinduction via demineralized bone which initiates a biological cascade resulting in endochondral ossification [37], [38]. In the current and previous studies, we describe the observed early, woven bone proliferation as intramembranous rather than endochondral [21], [22], [39], which better fits with Albrektsson and Johansson's definition. Taken one step further, we consider the relative barrier properties of the membrane sleeve, in conjunction with the impermeable surface of the intramedullary nail, to provide a conduit that guides bone regeneration inwards radially, similar (but in an opposite direction) to that suggested by Gogolewski and colleagues [40]. Finally, a gradient of cells and osteogenic factors is expected to develop between the inner surface of the directional delivery membrane and the outer surface of the IM nail, as well as between the edges and center of the defect zone; cellular and biochemical gradients can further facilitate osteogenesis via osteoinduction and osteoconduction.

Histomorphological studies are underway to elucidate quantitatively the time course of defect infilling via osteoinductive and osteoconductive mechanisms. In the previous study, in groups where periosteum, with small cortical bone chips adherent to the inner surface, was left *in situ* around critical sized defects, rapid proliferative woven bone was shown to infill the defect zone within two weeks of surgery (as evidenced by fluorochrome labeling during this time period) [21], [22]. In that study, by sixteen weeks after surgery, bone consolidated and its density and volume were shown to be highly modulated by the loading history to which the bone generated within the defect zone was subjected. Namely, in areas along the bone axis most able to resist bending loads (along the major centroidal axis), regenerate bone volume was higher and regenerate bone density was lower than in areas along the bone axis least able to resist bending (along the minor centroidal axis). Furthermore, at the conclusion of the previous study, it was impossible to assess wither bone induction or conduction had played a more important role in healing, although the rapid proliferative woven bone laid down in defects not packed with morcellized bone graft appeared to favor a more rapid maturation and remodeling of regenerate bone in the sixteen weeks of the experimental study [21], [22]. Of note, in the current study, the axial ingression (osteoconduction) was more apparent in the proximal half of the defect and was observed in the specimens examined at 16 weeks after surgery but not in those examined 3 weeks after surgery (where osteoinduction **was** observed radially from the implant toward the IM nail surface). An ongoing quantitative histomorphological analysis of the specimens from the current study should allow us to elucidate the spatiotemporal mechanisms of bone generation occurring radially via periosteum derived cells and axially via bone and periosteum of the proximal and distal bone segments.

Previously published studies have underscored the need to engineer periosteum substitutes [41] but have met with limited success [42], [43] in bridging critical sized defects and few have been tested in large animal models where scale up of cell mediated tissue generation is expected to be a major limitation. The approach employed in the current study capitalizes on osteoinductive and osteoconductive properties of periosteum substitutes, while providing a means for directional delivery of cells and osteoinductive factors inherent to the periosteum as well as modulation of the concentration of factors through tuning of the pore gradient on the inner membrane surface. In effect, this allows for vectorial delivery (a *vector* being defined by its magnitude and direction) of cells and osteoinductive factors, filling in the defect from the outside in, much like the mechanism by which osteoblasts infill resorption cavities during bone remodeling [32], [44].

There are several limitations inherent to the design, as well as to financial constraints, associated with *in vivo* studies in large animal models. First and foremost, we decided to retain the custom, stainless steel IM nail in the femoral tissue blocks at the conclusion of the study in order to retain the precise spatial organization of the biological samples. This was not necessary in our previous study using periosteal sleeves around the defect, because all defects were bridged and thus mechanically stable at the conclusion of the study. Retention of the nail had a further implication of not allowing for quantitative μ-CT analysis due to beam hardening and due to artifacts in imaging attributable to the presence of metallic implants [45]. However, histomorphometric study of femoral blocks that are fixed, embedded in PMMA, and sectioned serially *in toto* allowed for quantitation of tissue generation and comparison between groups. Ongoing histological analysis will allow for maximally precise reconstruction of spatial and temporal (though analysis of chelating fluorochromes administered at defined timepoints in the study) bone apposition in the sixteen weeks after surgery. Furthermore, given unlimited resources, it would have been desirable to include more experimental groups in the study to better elucidate effects of independent variables. Nonetheless, the current study provides a foundation that will help us to prioritize follow on studies, including *in silico* (virtual) models that allow for prediction of outcomes through variation of model parameters [46].

In trauma patients, as well as in surgical reconstruction patients, the amount of remaining healthy periosteum represents a limit to the use of the endogenous engineering approach demonstrated to bridge critical sized defects in the ovine femur (one-stage bone-transport procedure) [21], [22]. The current studies, implementing the newly developed periosteal substitutes, augmented through addition of periosteal transplant from other bone sites, test the use of the technology as a delivery vehicle for the patient's endogenous bone healing factors, expanding the indications for surgical reconstruction sheets in treatment of long bone defects. In addition, the periosteal substitutes, enhanced through seeding with auto- or allogenic stem cells, further expand the use of the technology for a range of tissue defects. The technology, which is based on a modular platform combining FDA approved materials, is poised for translation from the lab bench to the surgical patient.

## Supporting Information

Figure S1 Sixteen weeks after surgery. High resolution micro-computed tomography (μ-CT) images from the five femora making up the Control group, which was treated with an isotropic surgical membrane. Infilling occurs mainly through axial osteoconduction from proximal and distal edges toward the center of the defect zone.
(TIFF)

Figure S2 **A.** Three weeks after surgery. High resolution micro-computed tomography (μ-CT) images from the five femora making up Group 1, which was treated with the directional delivery membrane incorporating collagen sheets. Infilling occurs mainly radially, via inward intramembranous bone formation, from the inner surface of the surgical membrane towards the outer surface of the intramedullary nail. **B.** Sixteen weeks after surgery. High resolution micro-computed tomography (μ-CT) images from the five femora making up Group 1 of the current study, which was treated with the directional delivery membrane incorporating collagen sheets. Radial intramembranous bone formation observed at three weeks is no longer evident. Small amounts of infilling occur via axial osteoconduction from proximal and distal edges of the defect zone.
(TIFF)

Figure S3 Sixteen weeks after the two stage surgery with a directional delivery membrane incorporating collagen sheets seeded with autogenous periosteum-derived cells. High resolution micro-computed tomography (μ-CT) images of the femoral defect zones in the five femora making up Group 2. Infilling occurs radially via osteoinduction and axially via osteoconduction. Best infilling is observed in cases where the two coalesce.
(TIFF)

Figure S4 Sixteen weeks after the two stage surgery with a directional delivery membrane incorporating strips of autogenous periosteum from the bone removed to create the defect. High resolution micro-computed tomography (μ-CT) images of the femoral defect zones in the five femora making up Group 3. Infilling occurs radially via osteoinduction and axially via osteoconduction. Best infilling is observed in cases where the two coalesce.
(TIFF)
