# Supplementary figures and images for: Surgical Membranes as Directional Delivery Devices to Generate Tissue: Testing in an Ovine Critical Sized Defect Model
Source: PLoS One. 2011 Dec 12;6(12):e28702. doi: 10.1371/journal.pone.0028702 (PMC3236208; doi:10.1371/journal.pone.0028702)

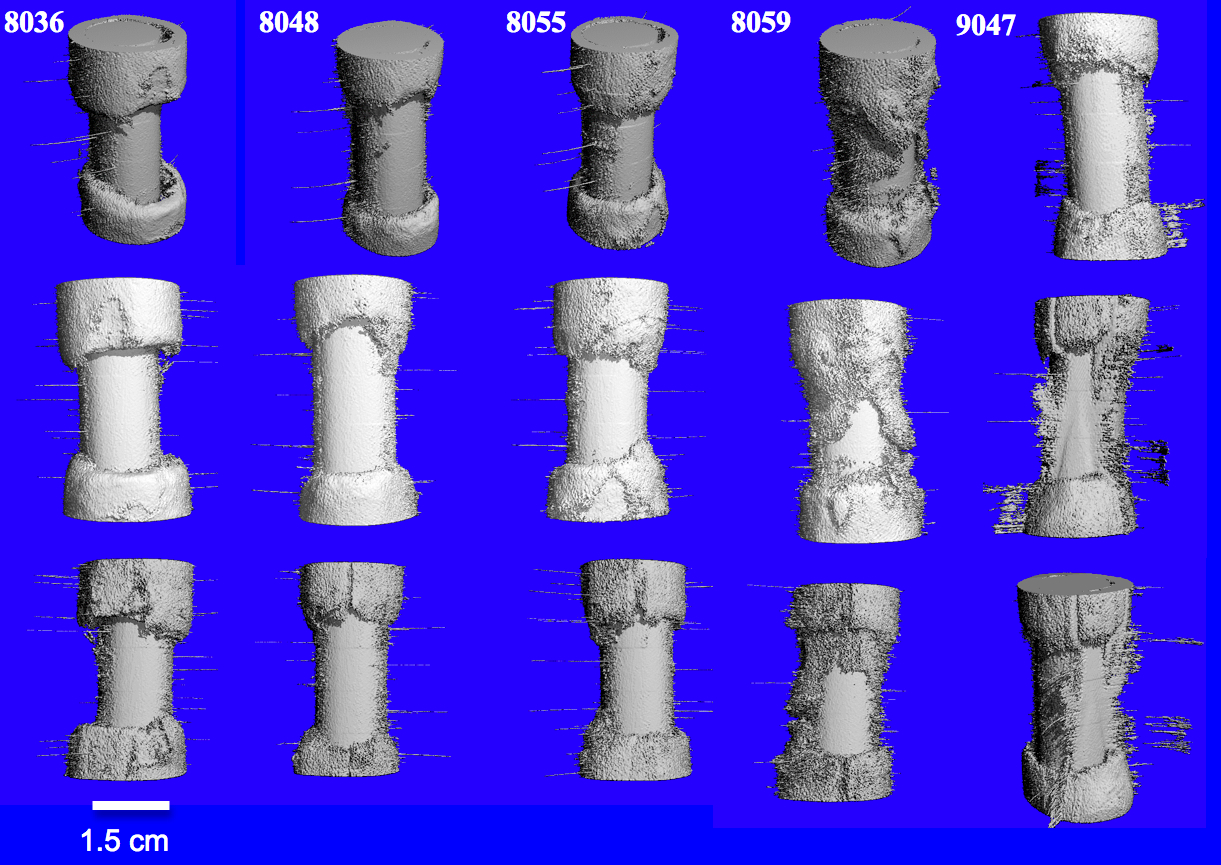

Supplement: Figure S1 — Sixteen weeks after surgery. High resolution micro-computed tomography (μ-CT) images from the five femora making up the Control group, which was treated with an isotropic surgical membrane. Infilling occurs mainly through axial osteoconduction from proximal and distal edges toward the center of the defect zone. (TIFF) [file pone.0028702.s001.tiff]

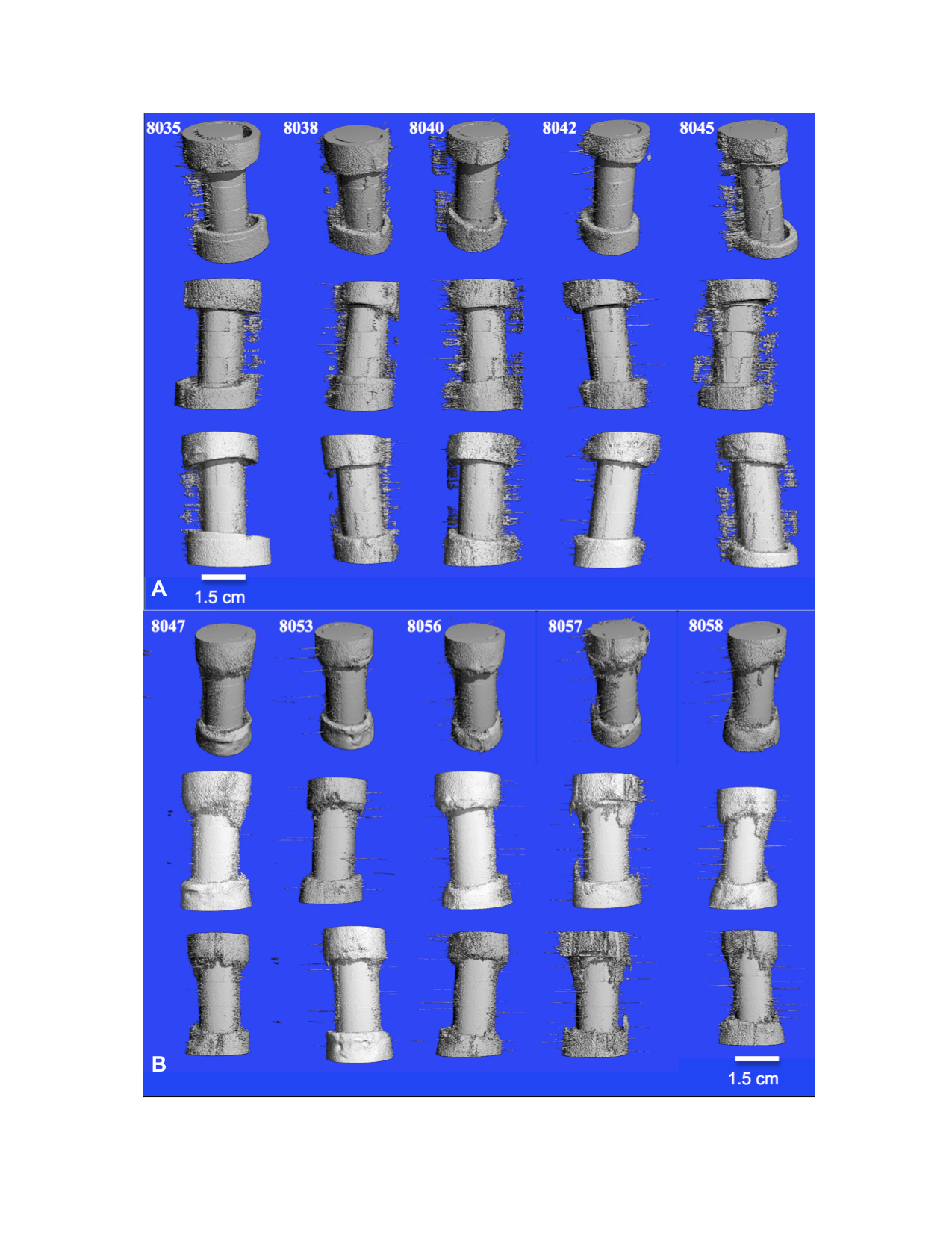

Supplement: Figure S2 — A. Three weeks after surgery. High resolution micro-computed tomography (μ-CT) images from the five femora making up Group 1, which was treated with the directional delivery membrane incorporating collagen sheets. Infilling occurs mainly radially, via inward intramembranous bone formation, from the inner surface of the surgical membrane towards the outer surface of the intramedullary nail. B. Sixteen weeks after surgery. High resolution micro-computed tomography (μ-CT) images from the five femora making up Group 1 of the current study, which was treated with the directional delivery membrane incorporating collagen sheets. Radial intramembranous bone formation observed at three weeks is no longer evident. Small amounts of infilling occur via axial osteoconduction from proximal and distal edges of the defect zone. (TIFF) [file pone.0028702.s002.tiff]

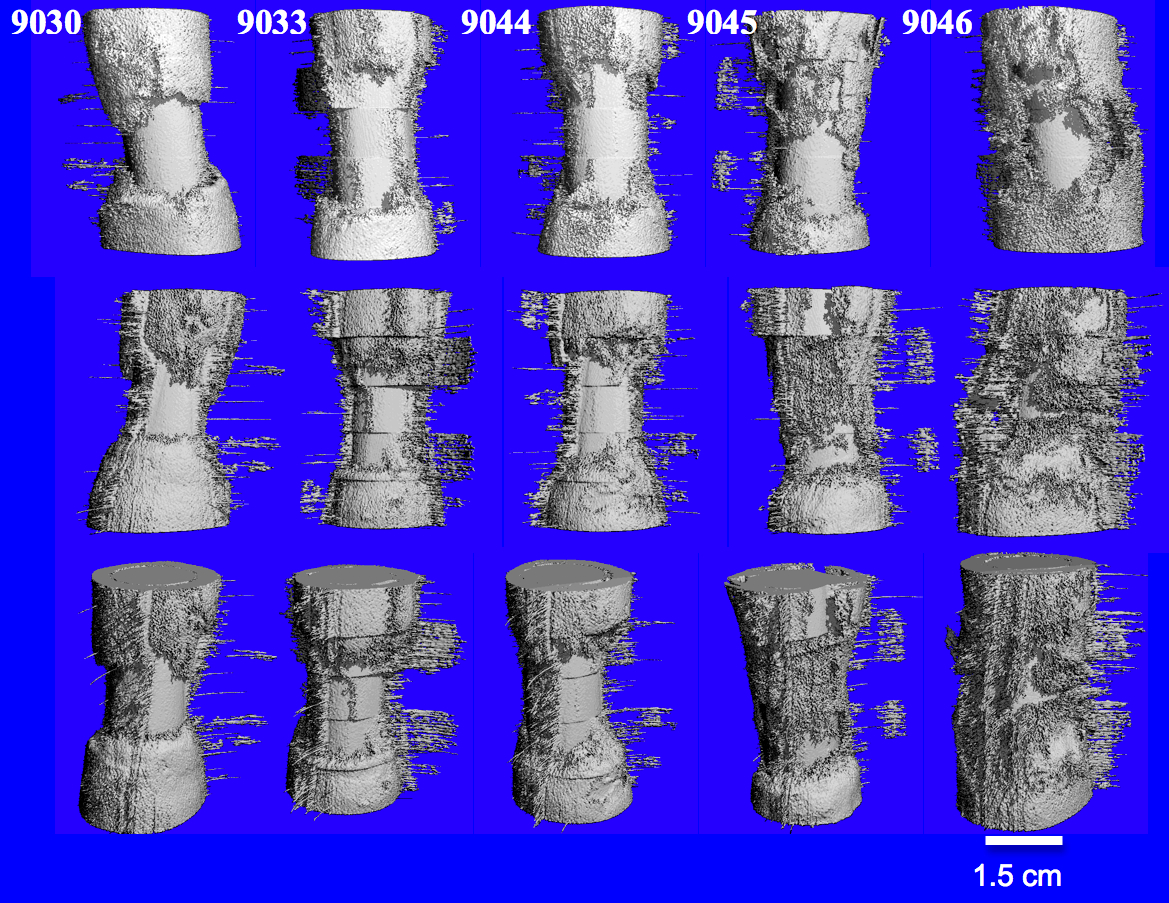

Supplement: Figure S3 — Sixteen weeks after the two stage surgery with a directional delivery membrane incorporating collagen sheets seeded with autogenous periosteum-derived cells. High resolution micro-computed tomography (μ-CT) images of the femoral defect zones in the five femora making up Group 2. Infilling occurs radially via osteoinduction and axially via osteoconduction. Best infilling is observed in cases where the two coalesce. (TIFF) [file pone.0028702.s003.tiff]

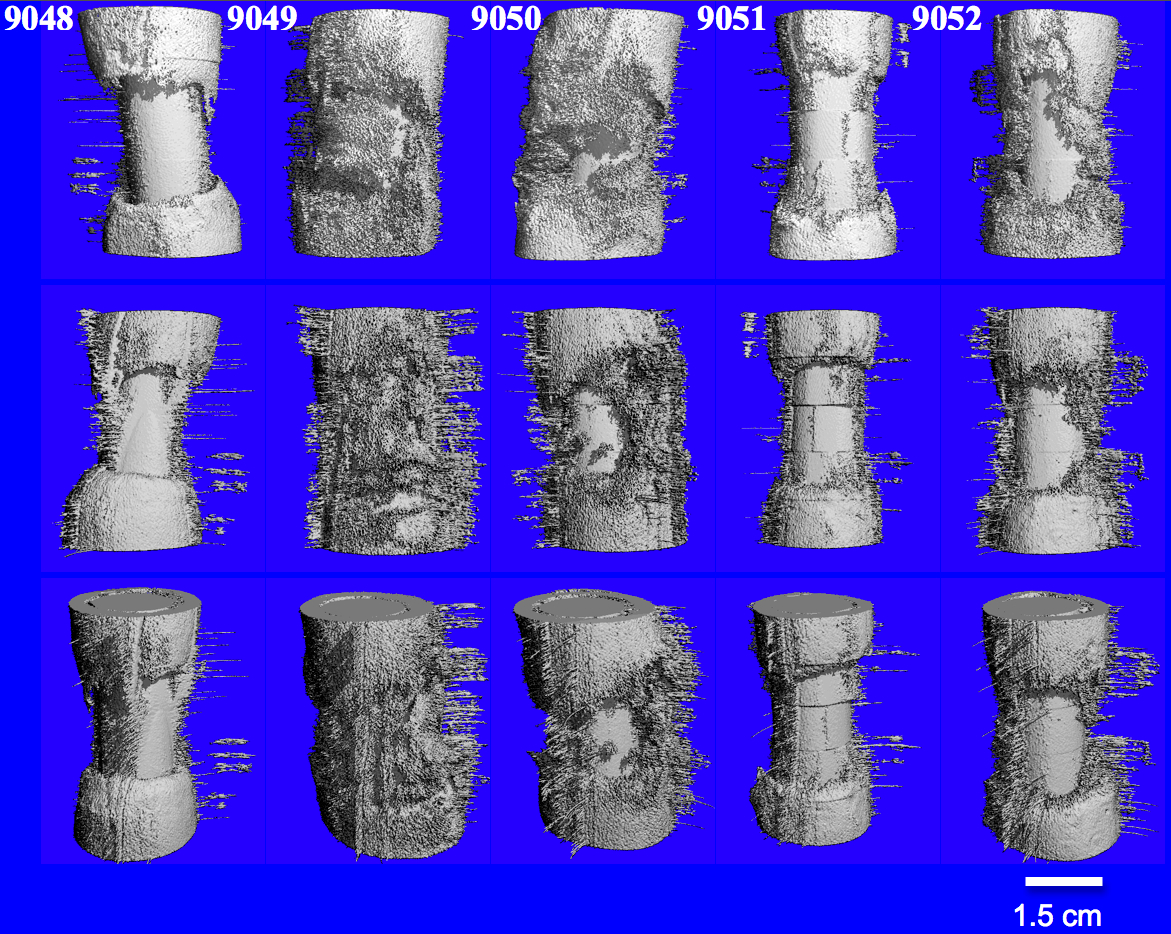

Supplement: Figure S4 — Sixteen weeks after the two stage surgery with a directional delivery membrane incorporating strips of autogenous periosteum from the bone removed to create the defect. High resolution micro-computed tomography (μ-CT) images of the femoral defect zones in the five femora making up Group 3. Infilling occurs radially via osteoinduction and axially via osteoconduction. Best infilling is observed in cases where the two coalesce. (TIFF) [file pone.0028702.s004.tiff]
